# Is Self-Incompatibility Related to Nectar Presence in *Dendrobium*?

**DOI:** 10.3390/plants14101496

**Published:** 2025-05-16

**Authors:** Karolina Zielińska, Kamil Kisło, Piotr Dobrzyński, Kevin L. Davies, Małgorzata Stpiczyńska

**Affiliations:** 1 Botanic Garden, Faculty of Biology, University of Warsaw, Al. Ujazdowskie 4, 00-478 Warsaw, Poland; 2 School of Earth and Environmental Sciences, Cardiff University, Main Building, Park Place, Cardiff CF10 3AT, UK; kevinldavies@btinternet.com

**Keywords:** Orchidaceae, nectar, nectaries, SI, SC, pollen-tubes

## Abstract

Studies on the gain or loss of nectar during the course of evolution in *Dendrobium* Sw. (Orchidaceae) are able to provide important information concerning the reproductive biology of this enormous orchid genus and highlight reproductive barriers—in particular, changes to compatibility. By employing a literature search on the compatibility system of *Dendrobium*, supplemented by new experimental data of 13 taxa investigated by means of microscopy, histochemistry, and phylogenetic analysis, we aimed to ascertain whether there is, in this genus, a relationship between self-compatibility (SC) and the presence of nectar. Nectariferous plant species are thought to be visited more frequently by pollinators, resulting in geitonogamy or selfing; therefore, the presence of nectar in some *Dendrobium* species may promote self-incompatibility (SI), whereas a lack of nectar may increase cross-pollination. Our investigations confirmed that the capacity for nectar secretion was gained and lost several times in this genus, and that similarly organized nectar spurs were present in all species investigated, regardless of their ability to produce nectar. SI, SC, and the presence or absence of nectar have all evolved independently, but, of the 42 taxa investigated whose status both relating to nectar presence and compatibility was known, nectar was more frequent in self-incompatible taxa.

## 1. Introduction

Orchids, like most flowering plants, require the service of pollinators for pollen transfer. Their flowers are known for their intricate adaptations to pollinators, as well as for their complex pollination biology and breeding systems. In orchids, pollinators gather a wide range of floral rewards, but nectar is reported to be the most common, providing pollinators with sugars, amino acids, lipids, and occasionally, secondary metabolites that can modify pollinator behavior [1,2,3,4]. Indeed, the literature reports that almost one-third of Orchidaceae are nectarless [5]. The flowers of many deceptive orchids are “empty” [6,7] and provide no reward whatsoever for visitors, even if they possess a spur resembling that found in nectariferous species.

It thus follows that the presence or absence of nectar, and its gain or loss during the course of evolution provides an important trait for determining pollinator visits, pollinator specificity, and the formation of pre-pollination reproductive barriers [8,9], together with the fragmented distribution of individuals (common in epiphytic orchids), and pollen limitation. Such barriers, in turn, can reduce gene flow in previously interbreeding populations. Self-incompatibility (SI) reduces the proportion of compatible crosses in the case of small populations, resulting in reduced fitness [10,11]. Studies involving the evolution of pre- and post-zygotic isolation of *Dendrobium* Sw. (Orchidaceae) and the effect of changes in compatibility states within this genus, were conducted by Pinheiro et al. [12] and Niu et al. [13].

Generally, two main categories of self-incompatibility occur in flowering plants: gametophytic self-incompatibility (GSI) and sporophytic self-incompatibility (SSI) [11,14,15,16,17]. With the exception of Apostasioideae, self-incompatibility occurs in all subfamilies of Orchidaceae [18,19]. GSI has been documented in Orchidaceae for 750 species, including 65 species of *Dendrobium*, for which self-incompatibility has been studied in most detail [13,20,21]. GSI acts when the SI phenotype of the pollen is determined by its own (haploid) S genotype, resulting in pollen-tube growth being typically blocked at various points along the transmitting tract or in the ovary. Sporophytic self-incompatibility (SSI) is related to self-pollen recognition on the stigma and is controlled by a complex locus (S-locus) with a variety of S-haplotypes that determine pollen and stigma specificity [13,18].

Pinheiro et al. [12] proposed that self-compatibility (SC) may be the ancestral breeding system in *Dendrobium* based on crossing experiments conducted previously by Wilfret [22] and Johansen [20], as SC occurs in *D. macrophyllum*, which was used to root the tree. Pinheiro et al. [12] also reported frequent transitions between SC and SI in different independent *Dendrobium* clades. They did not, however, state the direction of these transitions. Niu et al. [13] analyzed the distribution pattern of SI in the Asian *Dendrobium* clade and speculated that there may be many different, recently evolved SI determinants characteristic either of this genus, or of the entire family Orchidaceae.

*Dendrobium* is one of the largest genera in the family, with more than 1200 taxa described to date [23]. According to Burzacka-Hinz et al. [24,25], the convergent evolution of many floral traits in *Dendrobium sensu lato* may have resulted from adaptations to pollinators. Micromorphological analysis of the labellum, combined with phylogenetic tree analysis showed that labellar structures do not necessarily reflect phylogenetic relationships [24]. As previously mentioned, data on SI in this enormous genus represents the most complete set of SI data that we have available for Orchidaceae. Unfortunately, our knowledge concerning the presence of nectar in this genus, which is directly related to reproduction, is limited [26].

*Dendrobium* contains both nectariferous and nectarless species. Nectar, when present, is secreted and accumulated within the spur, formed by the fused basal parts of the labellum, column-foot, and sepals. Both the length of the spur and its nectar content are very variable within the genus [26].

Based on the data available on the compatibility system of *Dendrobium*, we aimed to ascertain the capacity of this genus to secrete nectar relative to this system. We hypothesize that SI taxa tend to be nectariferous. According to the cross-promotion hypothesis [27], the presence or absence of a floral reward are alternative solutions to a trade-off between pollination quality and quantity. Nectariferous species are visited more frequently, resulting in geitonogamy or selfing, and therefore, the presence of nectar in *Dendrobium* may promote SI, whereas a lack of nectar may increase cross-pollination.

## 2. Materials and Methods

Throughout this study, we follow the same circumscriptions of *Dendrobium* adopted by Burzacka-Hinz et al. [25] who, in their work, focused primarily on the nominal section, which they refer to as *Dendrobium sensu stricto*. However, they also refer to a second group which they term *Dendrobium sensu lato*. This encompasses both the nominal section and all species that have ever been part of it (e.g., species of *Diplocaulobium* (Rchb.f.) Kraenzl., *Flickingeria* A.D. Hawkes and *Epigeneium* Gagnep. which occupy basal branches alongside *Dendrobium sensu stricto*). *Dendrobium sensu lato* is a monophyletic group whose members, based on analysis of nuclear marker ITS, evolved from a common ancestor. Concerning the presence of nectar, we used data relating to 35 species of *Dendrobium* previously published in a paper by Jia and Huang, [26], which investigated species in an orchid breeding and conservation station in Malipo County, Wenshan Autonomous Prefecture, Yunnan Province, China, and these were supplemented by our own new data for the following taxa: *Dendrobium aphyllum* (Roxb.) C.E.C. Fisch., *Dendrobium bigibbum* Lindl., *Dendrobium* × *delicatum* (F.M. Bailey) F.M. Bailey, *Dendrobium farmeri* Paxton, *Dendrobium friedericksianum* Rchb.f., *Dendrobium glomeratum* H.J. Veitch ex Rob., *Dendrobium kingianum* Bidwill ex Lindl., *Dendrobium macrophyllum* A. Rich., *Dendrobium modestum* Rchb.f., *Dendrobium nobile* Lindl., *Dendrobium polytrichum* Ames, *Dendrobium secundum* (Blume) Lindl. ex Wall., and *Dendrobium trinervium* Ridl. These species were cultivated in the greenhouse of the Botanic Garden of the University of Warsaw, Poland. We are aware that under greenhouse conditions, the quantity and quality of nectar may differ significantly from the values that would be obtained during studies conducted in the natural environment. Therefore, in this work we have included data only on whether nectar is present or absent.

In order to determine the presence of nectar and nectariferous tissue, the flowers were analyzed on the first day of anthesis. We limited our observations to fresh flowers so as to avoid washing away any small amounts of nectar by the use of fixative solution. The flowers were cut longitudinally and both halves of the flower, together with the content of the spur, were investigated using a Nikon NZ 100 stereo-microscope (Nikon Instruments INC., Melville, NY, USA). Subsequently, spur nectaries were hand-cut transversely and examined using light microscopy, including Nomarski Differential Interference Microscopy (NDIM), allowing for more precise observations of surface secretion, or by means of fluorescence microscopy (Nikon Eclipse Ni-U, in conjunction with a Prior 200 w lamp Prior Scientific Instruments Ltd., Cambridge, UK), a UV-2B cube filter (330–380 nm excitation filter), a 400 nm (LP) dichroic mirror, and a 435 nm (LP) barrier filter to check for the possible autofluorescence of nectar. The nectary tissues were stained for general histology with toluidine blue O (TBO), and also checked for the presence of intracellular starch and lipids with IKI (iodine/potassium iodide solution) and Sudan IV, respectively. Micrometry and photomicrography were undertaken using a DS-Fi2 high-definition digital camera and NIS-Elements imaging software (Nikon) ver. D 5.11.00.

In analyzing a possible relationship between nectar secretion and compatibility systems, we mostly used the previously published data available on *Dendrobium* (Supplementary Table S1). Furthermore, for *D. farmeri*, which had previously been determined as SI, we analyzed pollen-tube growth in 20 self-pollinated flowers from two individual plants in order to ascertain the stage at which pollen-tube growth is blocked. The samples (five flowers each) were examined at days 1, 2, 6, and 11 following pollination. For *D.* × *delicatum*, we performed self- and cross-pollination on 20 flowers each (four individuals, with flowers assigned randomly relative to pollination mode within each inflorescence), so as to check the development and growth of pollen-tubes, and the development of capsules, as well as seed morphology. By means of light microscopy, we found, for 100 seeds in each of the ten samples per capsule, the number of correctly developed seeds containing an embryo.

We also investigated the effect of pollination on the life-span of the flower. In *D. farmeri*, the life-span of five self-pollinated flowers was compared with five non-pollinated flowers, as a control, whereas in *D.* × *delicatum*, for this comparison, we used each of 10 self- and cross-pollinated flowers, with 20 flowers as a control. Preparation of material for microscopic observations was performed according to the method by Niu et al. [13]. The percentage of correctly developed seeds in *D.* × *delicatum* following self- and cross-pollination was calculated with LM from 10 samples, each containing 100 seeds.

### 2.1. Phylogenetic Analysis

The Asian *Dendrobium* clade phylogenetic tree was constructed using the maximum likelihood method and the following markers: nrITS, matK, trnH-psbA spacer, rbcL (Genbank accession numbers for sequences used by us are provided as Supplementary Table S2). All sequences were aligned using the MUSCLE algorithm [28] as implemented in SeaView v. 5.0.5 software [29]. Non-conserved positions were trimmed using Gblocks v. 0.81 [30]. Sequences of all four regions were concatenated using FasconCAT v. 1.0 [31]. Suitable evolution models were calculated using ModelTest-NG v. 0.1.7 [32]. Each nucleotide position in the codon of matK and rbcL regions and every other whole region were treated separately. Finally, RaxML-NG v. 1.2.0 software [33] was used to calculate the maximum likelihood tree with 1000 bootstrap replications as support values. Both analyses were conducted on the CIPRES gateway [34]. The tree was visualized and all of the features were added using iTOL v. 6.8.1 [35]. The tree and alignment were deposited in FigShare doi: 10.6084/m9.figshare.28479989. We used *Epigeneium* as an outgroup since it represents one of the basal branches alongside *Dendrobium sensu stricto* [25].

### 2.2. Statistical Analysis

We used the chi square test as implemented in R software (ver. 4.3.2) [36] to test whether there were significant differences between the SI, SC, and the presence or absence of nectar. In order to estimate the transition rates between character states, we used a continuous-time Markov model implemented in R. To estimate the transition rates between states (the evolutionary speed of the transition from one state to another), we used corHMM::corHMM() [37], specifying an unordered model that allows free estimation of all possible transitions between states. We conducted this analysis on a subset of our data containing only the species for which we had full information on compatibility and nectar status (n = 42).

## 3. Results

**Figure 1:**
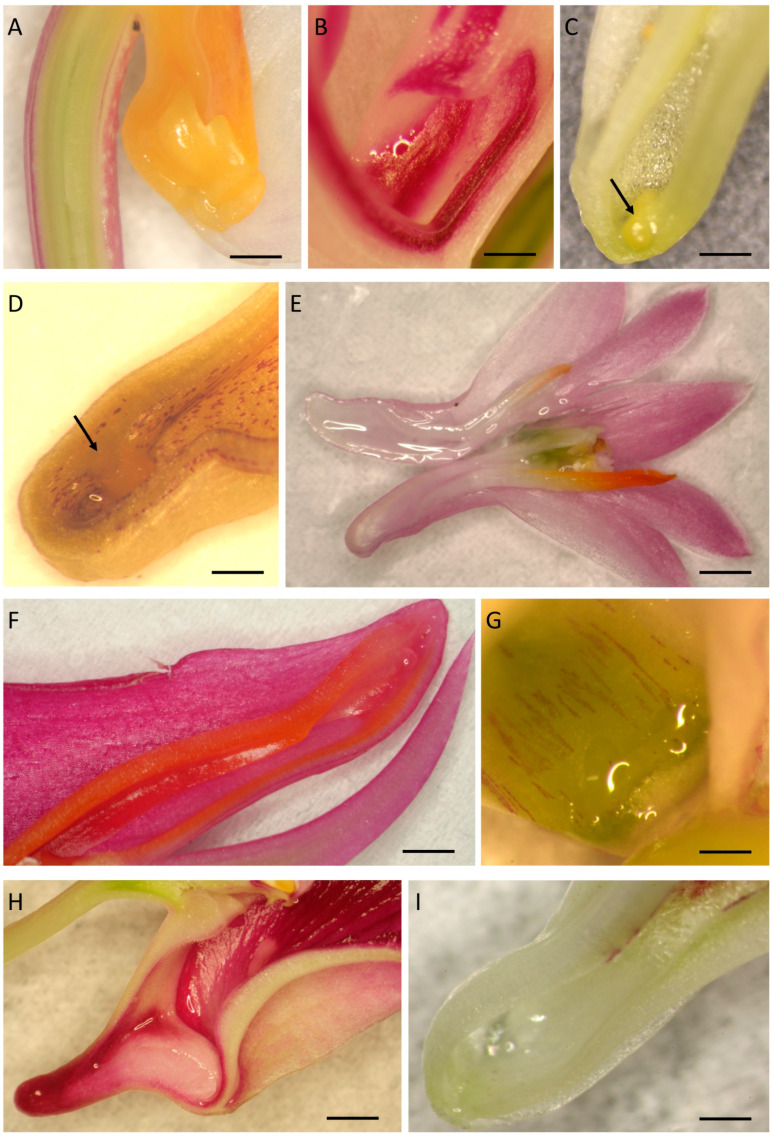
Morphology of nectary spurs. (**A**) *D. farmeri*; (**B**) *D. aphyllum*; (**C**) *D. modestum*; (**D**) *D. polytrichum*; (**E**) *D. secundum*; (**F**) *D. glomeratum*; (**G**) *D. kingianum*; (**H**) *D. bigibbum*; (**I**) *D. trinervium*. In (**C**,**D**), arrows indicate gland at the bottom of the spur. Scale bars: (**A**–**D**,**G**–**I**) = 1 mm; (**E**,**F**) = 5 mm.

**Figure 2:**
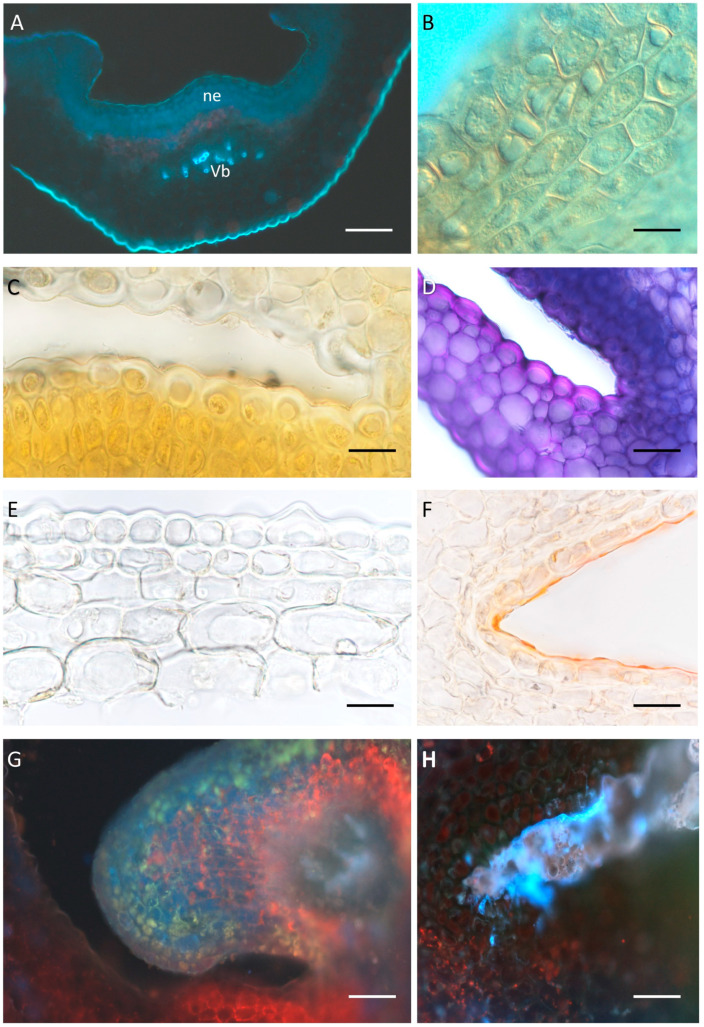
Anatomy of the nectaries. (**A**) Transverse section of the spur of *D. secundum* viewed in UV light, ne = nectary tissue; Vb = vascular bundles; (**B**) nectary tissue of *D. farmeri*, NDIM; (**C**,**D**) nectary tissue of *D. secundum* treated with IKI and TBO, respectively. Note thick cell walls; (**E**) section of the spur of nectarless *D.* x *delicatum* with thick-walled epidermal cells and relatively large subepidermal cells; (**F**) thin cuticle overlying epidermal cells in *D. nobile*; (**G**,**H**) *D. modestum*, UV light. (**G**) Note blue autofluorescence in secretory cells; (**H**) autofluorescence of nectar. Scale bars: (**A**) = 100 µm; (**B**–**F**) = 20 µm; (**G**,**H**) = 50 µm.

Macroscopic observations revealed the presence of nectar in *D. farmeri*, *D. aphyllum*, *D. modestum*, *D. polytrichum*, *D. secundum*, *D. glomeratum*, *D. kingianum*, *D. bigibbum*, *D. trinervium* (Figure 1A–I) and *D. macrophyllum*, whereas *D. nobile*, *D.* × *delicatum*, and *D. friedericksianum* were nectarless. In *D. modestum* and *D. polytrichum*, a small nectariferous gland was present at the bottom of the spur (Figure 1C,D), which was absent from the remaining investigated species. Nectary tissue was composed of several layers of cells, which were more numerous on the adaxial side of the spur (Figure 2A–D). Vascular bundles ran just below the secretory parenchyma (Figure 2A). In both nectariferous and nectarless species, the spur was lined with thick-walled epidermal cells (Figure 2C–F) bearing a thin cuticle (Figure 2F). The thickest cell walls were observed in *D. secundum* (mean = 8.45 µm), whereas the thinnest were found in *D. trinervium* (mean = 6.23 µm). Thick cell walls also occurred in the subepidermal parenchyma. In nectariferous species, secretory cells typically contained dense cytoplasm and relatively large nuclei (Figure 2B), but in nectarless species, the subepidermal tissue resembled ground parenchyma (Figure 2E,F). Storage starch was absent from the spur cells of investigated species, with the sole exception of *D. trinervium*, where it was present both in the epidermis and parenchyma. Only in the very fragrant *D. modestum* did nectar show blue autofluorescence under UV light, and weak blue autofluorescence was observed for cells of the nectary gland (Figure 2G,H).

**Figure 3:**
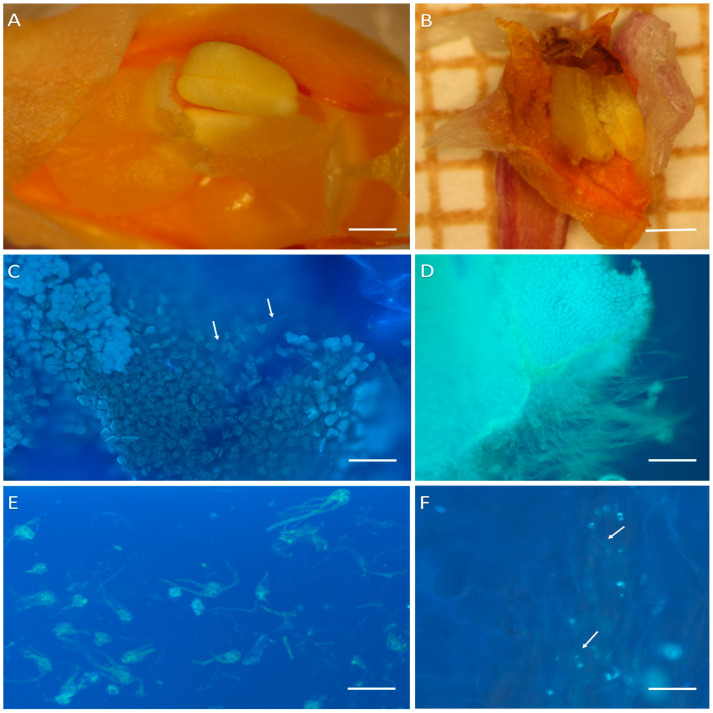
Self-pollination of *D. farmeri*. (**A**,**B**) Pollinia in the stigmatic chamber 2 and 11 days after pollination, respectively; (**C**) germinating pollen tubes (arrows) two days post-pollination; (**D**) pollinium and pollen tubes 11 days following pollination; (**E**) pollen tetrads and pollen tubes six days after pollination; (**F**) pollen tubes (arrows) in the upper part of stylar canal 11 days after pollination. Scale bars: (**A**) = 0.5 mm; (**B**) = 1 mm; (**C**,**E**,**F**) = 20 µm; (**D**) = 50 µm.

Self-pollinated flowers of *D. farmeri* began to wilt three days following pollination, and had completely dropped two weeks later. Non-pollinated flowers remained fresh for ten days longer. Concerning pollen-tube growth, pollinia became closely attached to the stigmatic chamber one day following self-pollination, and after two days their rehydration was evident (Figure 3A). At day 11, the perianth had completely wilted, and the stigmatic chamber had closed, but the pollinia remained attached to it (Figure 3B). Pollen germination was visible two days following pollination (Figure 3C) and stages in the growth of pollen-tubes were observed on days 6 and 11 (Figure 3D,E). On day 11, pollen-tubes were visible in the upper part of the stylar canal (Figure 3F) and were blocked at this level. We did not observe pollen-tubes in the ovary.

**Figure 4:**
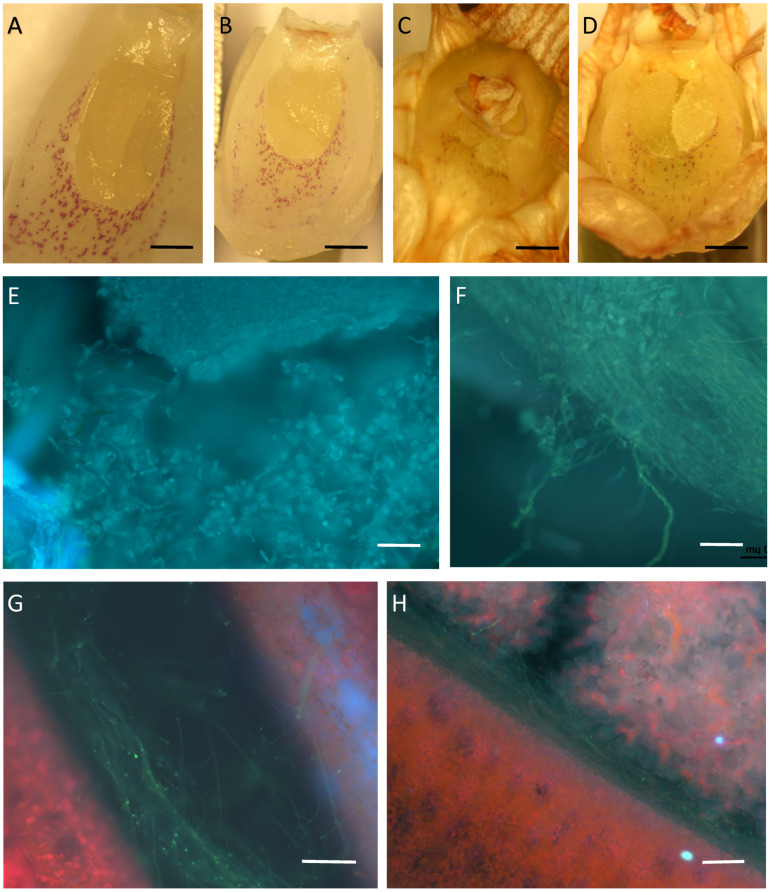
Stigmatic surface and pollinia in *D.* × *delicatum* at (**A**) 2, (**B**) 6 and (**C**) 11 days following self-pollination; (**D**) 11 days following cross pollination; (**E**) developing pollen tubes 2 days following cross pollination; (**F**) pollen tubes in stylar canal 6 days following pollination; (**G**,**H**) at 11 days following self-pollination, pollen-tubes are present in ovary (**G**) and among ovules (**H**). Scale bars: (**A**–**D**) = 0.5 mm; (**E**–**H**) = 20 µm.

**Figure 5:**
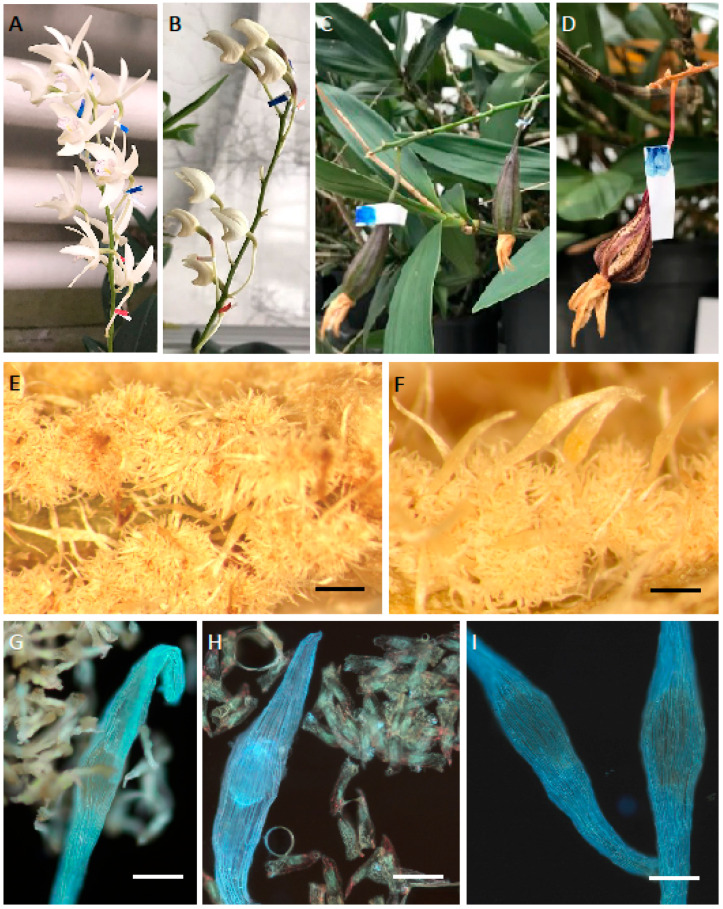
Fruit and seed of *D*. × *delicatum* following self- and cross-pollination. (**A**) Inflorescence with tagged self- and cross-pollinated flowers. (**B**) Seven days following pollination, the ovaries are obviously enlarged compared with non-pollinated flowers. The flowers from the middle part of the inflorescence were sampled for analysis; (**C**,**D**) ovaries at two and three months, respectively, following cross-pollination; (**E**,**F**) seed in capsules after self- and cross-pollination, respectively; (**G**,**H**) seed with and without embryo after self- and cross-pollination, respectively; (**I**) Seed with embryo after cross-pollination. Scale bars: (**E**,**F**) = 0.5 mm; (**G**–**I**) = 40 µm.

In *D.* × *delicatum* (Figure 4A–H and Figure 5A–I), there was no difference in the life-span of the flower, be it self- or cross-pollinated. Furthermore, its flowers, and perianth remained fresh for seven days less compared with non-pollinated flowers (Figure 5A,B). Neither did we observe any difference in the time required for the rehydration of pollinia and pollen-tube growth in self- and cross-pollinated flowers. Pollinia became closely attached to the stigmatic chamber two days following pollination. By the sixth day, the pollinia were completely rehydrated, and by day 11, the stigmatic chamber had shut, but the pollinia remained attached to it, irrespective of whether the flowers had been self- or cross-pollinated (Figure 4A–D). The development of pollen tubes began two days following pollination (Figure 4E) and by day 6, pollen tubes were present in the upper part of the stylar canal (Figure 4F). By day 11, they had reached the ovary and were observed both in the placenta and between the ovules (Figure 4G,H). Of the ten self-pollinated flowers, only two fruit had reached the ripening stage three months following pollination, whereas in the ten flowers that had been cross-pollinated, only one capsule reached maturity (Figure 5C,D). The remaining pollinated flowers, together will all the non-pollinated flowers, finally became senescent and dropped. In self-pollinated flowers, 5.3% of seed contained in the capsules had developed embryos, whereas in cross-pollinated flowers, this was 6.0% (Figure 5E–I).

**Figure 6:**
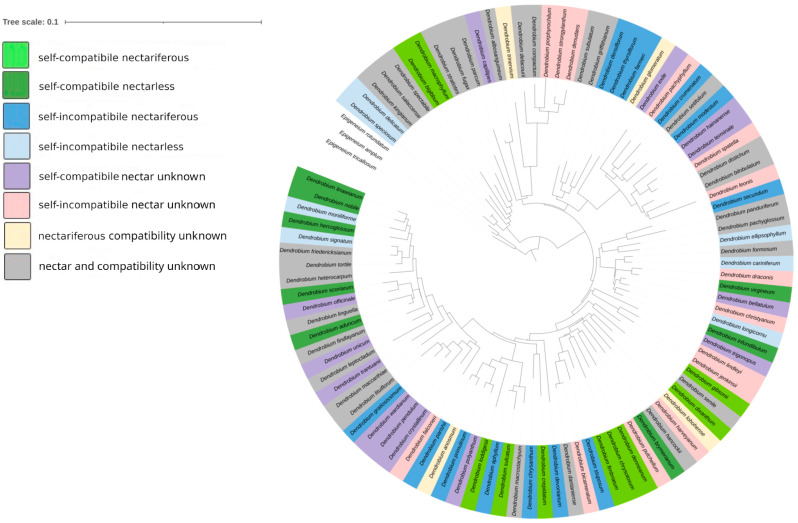
Phylogenetic tree of selected species of *Dendrobium*. The various colors indicate different combinations of self-compatibility and nectar presence in these species. Three species of the genus *Epigeneium* Gagnep. were used as the outgroup.

**Figure 7:**
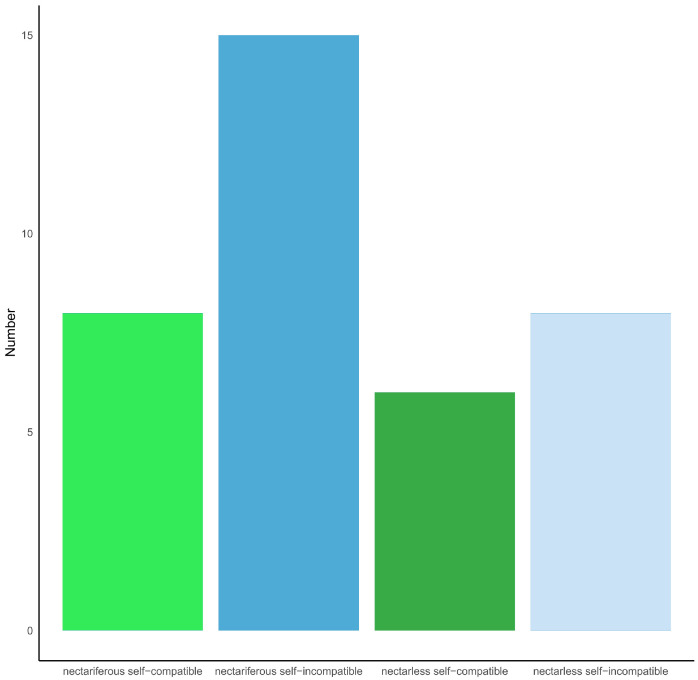
Number of nectariferous and nectarless SC and SI species of *Dendrobium*.

Phylogenetic analyses did not show any evolutionary trend relating to the presence of nectar and compatibility in *Dendrobium* spp. Self-compatibility, self-incompatibility, the capacity to secrete nectar and the absence of nectar, all evolved independently in the investigated taxa, within clades (Figure 6). However, of the 42 taxa investigated, whose status, both relating to nectar presence and compatibility, was known, nectar was more frequent in self-incompatible taxa (16 of the 23 investigated species); however, in 19 of the self-compatible taxa, since 10 species were nectariferous, this character was distributed almost evenly (chi square test: X^2^ = 0.020073, *p* > 0.05; Figure 7). The plesiomorphic condition in the analyzed representatives of Asian *Dendrobium* was SC and the presence of nectar. Our further analysis showed two state transitions, with the highest ratio occurring in nectarless species, namely a transition from SI nectarless to SC nectarless (474.80), and SC nectariferous to SC nectarless (36.89). Conversely, the transition ratio in nectarless taxa from SC to SI was negligible, whereas the ratio of transition from SC nectarless to SC nectariferous was 25.4. These values represent the evolutionary rates at which taxa shift from one discrete state to another over time, indicating the likelihood and directionality of trait changes for the given phylogenetic context.

## 4. Discussion

Our investigations confirmed that nectar secretion in *Dendrobium* was gained and lost several times during the course of evolution and that there were transitions, but we did not observe any obvious evolutionary trends in the latter. Similar results were also reported for *Dendrobium* species investigated by Lia and Huang [26], and for the large Neotropical genus *Epidendrum* [38]. In large genera such as these, whose species differ considerably in terms of morphology, habitats, and distribution, it is expected that flowers will display various strategies for attracting pollinators.

The presence of a nectary spur is typical for *Dendrobium*, irrespective of the presence of nectar [26], but it would appear that the capacity to secrete nectar can be modified relatively easily in response to the demands of pollinators. Histologically, the spurs of nectariferous and nectarless species were similar, but in active secretory tissue, the nectary cells had dense cytoplasm. Starch was generally absent from the cells of the spur of the investigated species, with the exception of *D. trinervium*. Starch is usually common in the nectary tissue, and this polysaccharide can serve as raw material for nectar sugars. The amount of starch present can vary from species to species, as has been shown for *Sobralia* and *Epidendrum* [39,40]. However, the presence of starch cannot be used as a predictor of nectary activity. For this investigation, we employed flowers at day 1 of anthesis, and therefore, the possibility cannot be excluded that starch had been used as a source of nectar-sugars or for fragrance synthesis during the pre-anthesis stage.

Our experiment confirmed self-incompatibility in *D. farmeri*, where growth of pollen-tubes was blocked in the stylar canal, but contrary to observations by Zang et al. [18], pollen required as much as 11 days to reach here. By contrast, in *D.* × *delicatum*, as in *Bulbophyllum* [41], pollen-tubes had reached the ovary by approximately day 11 following pollination. However, most pollinated flowers were aborted, and only a few well-developed seed were found in the capsules, regardless of whether the flowers were self- or cross-pollinated. It is possible that poor seed-set may have been due to the fact that this taxon is a natural hybrid (*D. kingianum* × *D. speciosum* var. *hillii*), and *D. speciosum* is itself self-incompatible [18]. Unfortunately, there is not much data in the literature on seed quality in *Dendrobium* species. Pinhero et al. [12] reported values related to seed-set ranging from 10.68% to 42.86% based on data obtained by Wilfret [22] and Johansen [20], but they refer to the interspecific crosses.

Although in some clades the presence of both nectar and compatibility appear to be conserved (e.g., in the *Dendrobium strongylanthum* clade), our analyses show that these characters evolved separately in each *Dendrobium* clade. Some sister species have the same reproduction strategy (e.g., *D. nobile* and *D. linawianum*), whereas other sister species have contrasting self-compatibility and nectar-secreting status (e.g., *D. aphyllum* and *D. sulcatum*). Consequently, it is not possible to predict these characters for *Dendrobium* flowers based solely on the phylogenetic position of a specific taxon. Neither our research nor published data [12,26], were sufficient to trace evolutionary trends in *Dendrobium*, particularly since we were able to consider only the 42 taxa for which we knew full compatibility and nectar data. Although the most basal clade (*D. macrophyllum*) is nectariferous and self-compatible, other clades contain mostly species of unknown reproductive strategy. Therefore, we were not able to reach conclusions regarding the ancestral characters of *Dendrobium*.

Studies involving the reconstruction of the ancestral states of *Dendrobium sensu lato* by Burzacka-Hinz et al. [25], however, showed that most characters relating to vegetative morphology, flowers and inflorescences arose independently several times as a result of convergent evolution. Consequently, the possibility cannot be excluded that the capacity for nectar secretion and compatibility has also evolved completely independently in this genus, thus optimizing its reproductive success. Even though our analyses showed that nectar secretion was more frequently associated with self-incompatibility, as our hypothesis had predicted, our test showed two state transitions, the highest ratio occurring from SI nectarless to SC nectarless (and therefore independent of nectar presence), and from SC nectariferous to SC nectarless. The highest transition ratio from the nectariferous to nectarless state may indicate a tendency towards resource-saving while still maintaining pollinator service. However, due to the relative paucity of data for this large genus, we must approach these conclusions with great caution, as well as conclusions concerning evolutionary trends relating to nectar secretion and compatibility systems. Nevertheless, our studies may indicate the direction that further research ought to take in future, and this, in turn, by providing additional information on nectar secretion and the compatibility status of *Dendrobium*, should contribute to our general understanding of the evolution of this enormous genus.

## Data Availability

Data is provided within the manuscript. All additional data supporting the presented results are included as Supplementary Files.

## Supplementary Materials

The following supporting information can be downloaded at: https://www.mdpi.com/article/10.3390/plants14101496/s1, Table S1: Literature data on compatibility status and nectar secretion in *Dendrobium*. Table S2: Genbank accession numbers.

## References

[1] Roy R., Schmitt A.J., Thomas J.B., Carter C.J. (2017). Nectar biology: From molecules to ecosystems. Plant Sci..

[2] Nicolson S.W. (2022). Sweet solutions: Nectar chemistry and quality. Philos. Trans. R. Soc. B.

[3] Göttlinger T., Lohaus G. (2022). Comparative analyses of the metabolite and ion concentrations in nectar, nectaries, and leaves of 36 bromeliads with different photosynthesis and pollinator types. Front. Plant Sci..

[4] Barberis M., Calabrese D., Galloni M., Nepi M. (2023). Secondary metabolites in nectar-mediated plant-pollinator relationships. Plants.

[5] Shrestha M., Dyer A.G., Dorin A., Ren Z.-X., Burd M. (2020). Rewardlessness in orchids: How frequent and how rewardless?. Plant Biol..

[6] Cozzolino S., Aceto S., Caputo P., Widmer A., Dafni A. (2001). Speciation processes in Eastern Mediterranean Orchis s.l. species: Molecular evidence and the role of pollination biology. Isr. J. Plant Sci..

[7] Johnson S.D., Hobbhahn N., Bytebier B. (2013). Ancestral deceit and labile evolution of nectar production in the African orchid genus *Disa*. Biol. Lett..

[8] Cozzolino S., Scopece G. (2008). Specificity in pollination and consequences for postmating reproductive isolation in deceptive Mediterranean orchids. Philos. Trans. R. Soc. B.

[9] Pinheiro F., de Melo e Gouveia Z., Manuel T., Cozzolino S., Cafasso D., Cardoso-Gustavson P., Suzuki R.M., Palma-Silva C. (2016). Strong but permeable barriers to gene exchange between sister species of *Epidendrum*. Am. J. Bot..

[10] Byers D.L., Meagher T.R. (1992). Mate availability in small populations of plant-species with homomorphic sporophytic self-incompatibility. Heredity.

[11] Brandvain Y., Haig D. (2005). Divergent mating systems and parental conflict as a barrier to hybridization in flowering plants. Am. Nat..

[12] Pinheiro F., Cafasso D., Cozzolino S., Scopece G. (2015). Transitions between self-compatibility and self-incompatibility and the evolution of reproductive isolation in the large and diverse tropical genus *Dendrobium* (Orchidaceae). Ann. Bot..

[13] Niu S.-C., Huang J., Xu Q., Li P.-X., Yang H.-J., Zhang Y.-Q., Zhang G.-Q., Chen L.-J., Niu Y.-X., Luo Y.-B. (2018). Morphological type identification of self-incompatibility in *Dendrobium* and its phylogenetic evolution pattern. Int. J. Mol. Sci..

[14] Nettancourt D. (2001). Incompatibility and Incongruity in Wild and Cultivated Plants.

[15] Barrett S.C.H. (2013). The evolution of plant reproductive systems: How often are transitions irreversible?. Proc. Biol. Sci..

[16] Ivano M., Takayama S. (2012). Self/non-self discrimination in angiosperm self-incompatibility. Curr. Opin. Plant Biol..

[17] Niu S., Huang J., Zhang Y., Li P., Zhang G., Xu Q. (2017). Lack of S-RNase based gametophytic self-incompatibility in orchids suggests that this system evolved after the monocot-eudicot split. Front. Plant Sci..

[18] Zhang X., Jia Y., Liu Y., Chen D., Luo Y., Niu S. (2021). Challenges and perspectives in the study of self-incompatibility in orchids. Int. J. Mol. Sci..

[19] Ricci N.A.P., Bento J.P.S.P., Mayer J.L.S., Singer R.B., Koehler S. (2004). Gametophytic self-incompatibility in Maxillariinae orchids. Protoplasma.

[20] Johansen B. (1990). Incompatibility in *Dendrobium* (Orchidaceae). Bot. J. Linn. Soc..

[21] Pinheiro F., Cardoso-Gustavson P., Suzuki R.M., Abrao M.C.R., Guimaraes L.R., Draper D., Moraes A.P. (2015). Strong postzygotic isolation prevents introgression between two hybridizing Neotropical orchids, *Epidendrum denticulatum* and *E. fulgens*. Evol. Ecol..

[22] Wilfret G.J., Kamemoto H. (1969). Genome and Karyotype Relationships in the Genus *Dendrobium* (Orchidaceae). I. Crossability. Am. J. Bot..

[23] Schuiteman A., Adams P.B., Pridgeon A.M., Cribb P.J., Chase M.W., Rasmussen F.N. (2014). *Dendrobium*-Infrageneric treatment. Genera Orchidacearum, Epidendroideae (Part Three).

[24] Burzacka-Hinz A., Narajczyk M., Dudek M., Szlachetko D.L. (2022). Micromorphology of labellum in selected *Dendrobium* Sw. (Orchidaceae, Dendrobieae). Int. J. Mol. Sci..

[25] Burzacka-Hinz A., Dudek M., Olędrzyńska N., Naczk A.M., Szlachetko D.L. (2025). Evolution of morphological traits of *Dendrobium* sensu lato (Orchidaceae)—An attempt to resolve phylogenetic relationships in nominal and morphologically convergent sections. BMC Plant Biol..

[26] Jia B., Huang S.-Q. (2021). An examination of nectar production in 34 species of *Dendrobium indicates* that deceptive pollination in the orchids is not popular. J. Syst. Evol..

[27] Peakall R., Beattie A.J. (1996). Ecological and Genetic Consequences of Pollination by Sexual Deception in the orchid *Caladenia tentaculata*. Evolution.

[28] Edgar R.C. (2004). MUSCLE: Multiple Sequence Alignment with High Accuracy and High Throughput. Nucleic Acids Res..

[29] Galtier N., Gouy M., Gautier C. (1996). SEAVIEW and PHYLO_WIN: Two Graphic Tools for Sequence Alignment and Molecular Phylogeny. Bioinformatics.

[30] Castresana J. (2000). Selection of conserved blocks from multiple alignments for their use in phylogenetic analysis. Mol. Biol. Evol..

[31] Kück P., Meusemann K. (2010). FASconCAT: Convenient handling of data matrices. Mol. Phylogenet. Evol..

[32] Darriba D., Posada D., Kozlov A.M., Stamatakis A., Morel B., Flouri T. (2020). ModelTest-NG: A New and scalable tool for the selection of DNA and protein evolutionary models. Mol. Biol. Evol..

[33] Kozlov A.M., Darriba D., Flouri T., Morel B., Stamatakis A. (2019). RAxML-NG: A Fast, scalable and user-friendly tool for Maximum Likelihood Phylogenetic Inference. Bioinformatics.

[34] Miller M.A., Pfeiffer W., Schwartz T. Creating the CIPRES Science Gateway for Inference of Large Phylogenetic Trees. Proceedings of the 2010 Gateway Computing Environments Workshop (GCE).

[35] Letunic I., Bork P. (2024). Interactive Tree of Life (iTOL) v6: Recent updates to the phylogenetic tree display and annotation tool. Nucleic Acids Res..

[36] R Core Team (2023). R: A Language and Environment for Statistical Computing.

[37] Beaulieu J.M., O’Meara B.C., Donoghue M.J. (2013). Identifying Hidden Rate Changes in the Evolution of a Binary Morphological Character: The Evolution of Plant Habit in Campanulid Angiosperms. Syst. Biol..

[38] Cardoso-Gustavson P., Saka M.N., Pessoa E.M., Palma-Silva C., Pinheiro F. (2018). Unidirectional transitions in nectar gain and loss suggest food deception is a stable evolutionary strategy in *Epidendrum* (Orchidaceae): Insights from anatomical and molecular evidence. BMC Plant Biol..

[39] Neubig K.M., Carlsward B.S., Whitten M.W., Williams N.H. (2015). Nectary structure and nectar in *Sobralia* and *Elleanthus* (Sobralieae: Orchidaceae). Lankesteriana.

[40] Stpiczyńska M., Kamińska M., Davies K.L., Pansarin E.R. (2018). Nectar-secreting and nectarless *Epidendrum*: Structure of the inner floral spur. Front. Plant Sci..

[41] Rodrigues J.G., Borba E.L. (2023). Variation in self-incompatibility and interspecific compatibility in a lineage of the mostly self-compatible genus *Bulbophyllum* (B. sect. Micranthae–Orchidaceae). Plant Syst. Evol..

