# Supplementary material for: Is Self-Incompatibility Related to Nectar Presence in Dendrobium?
Source: Plants (Basel). 2025 May 16;14(10):1496. doi: 10.3390/plants14101496 (PMC12115144; doi:10.3390/plants14101496)
Supplement: Supplementary file 1 [file plants-14-01496-s001.zip › Supplementary Table S1. Literature data on compatibility system and nectar secretion i Dendrobium.pdf]

Supplementary Table S1. Literature data on compatibility status and nectar secretion in *Dendrobium*.

| **Species** | **SI/ SC**[i] | **Dataset** | **N/NL**[ii] | **Dataset** |
|---|---|---|---|---|
| *D. acinaciforme* Roxb. | SI | Pinheiro et al. 2015 | | |
| *D. aduncum* Lindl. | SC | Niu et al. 2018 | NL | Jia, Huang 2021 |
| *D. aggregatum* Kunth | SI | Pinheiro et al. 2015 | | |
| *D. albosanguineum* Lindl. & Paxton | SI | Pinheiro et al. 2015 | | |
| *D. aloifolium* (Blume) Rchb.f. | SI | Pinheiro et al. 2015 | | |
| *D. alterum* Seidenf. | SI | Pinheiro et al. 2015 | | |
| *D. anosmum* Lindl. | - | - | N | Jia, Huang 2021 |
| *D. aphyllum* (Roxb.) C.E.C.Fisch. | SI | Niu et al. 2018<br>Pinheiro et al. 2015 | N | Jia, Huang 2021 |
| *D. arachnites* Thouras | SI | Pinheiro et al. 2015 | | |
| *D. bellatulum* Rolfe | SC | Niu et al. 2018 | | |
| *D. bicameratum* Lindl. | SI | Niu et al. 2018<br>Pinheiro et al. 2015 | | |
| *D. bigibbum* Lindl. | SC | Pinheiro et al. 2015 | N | Own research |
| *D. bilobulatum* Seidenf. | SC/SI | Pinheiro et al. 2015 | - | - |
| *D. brymerianum* Rchb.f. | SC | Niu et al. 2018<br>Pinheiro et al. 2015 | NL | Jia, Huang 2021 |
| *D. capillipes* Rchb.f. | SC | Niu et al. 2018 | | |
| *D. cariniferum* Rchb.f. | SC | Niu et al. 2018 | NL | Jia, Huang 2021 |
| *D. christyanum* Rchb.f. | SI | Niu et al. 2018<br>Pinheiro et al. 2015 | | |
| *D. chrysanthum* Wall. ex Lindl. | SI | Niu et al. 2018 | N | Jia, Huang 2021 |
| *D. chrysotoxum* Lindl. | SC | Niu et al. 2018 | N | Jia, Huang 2021 |
| *D. compactum* Rolfe ex Hemsl. | SI | Pinheiro et al. 2015 | | |
| *D. concinnum* Miq. | - | - | | |
| *D. crepidatum* Griff. | SC | Niu et al. 2018<br>Pinheiro et al. 2015 | N | Jia, Huang 2021 |
| *D. crumenatum* Sw. | SI | Niu et al. 2018<br>Pinheiro et al. 2015 | N | Jia, Huang 2021 |

| | | | | |
|---|---|---|---|---|
| *D. crystallinum* Rchb.f. | SC | Niu et al. 2018 | | |
| *D. delacourii* Guillaumin | SC | Pinheiro et al. 2015 | | |
| *D.delicatum* (F.M. Bailey) F.M. Bailey | SI | Own research | NL | Own research |
| *D. denneanum* Kerr | SC | Niu et al. 2018 | N | Jia, Huang 2021 |
| *D. densiflorum* Lindl. | SI | Niu et al. 2018 | N | Jia, Huang 2021 |
| *D. denudans* D.Don. | SI | Niu et al. 2018 | | |
| | | Pinheiro et al. 2015 | | |
| *D. devonianum* Paxton | SI | Niu et al. 2018 | N | Jia, Huang 2021 |
| | | Pinheiro et al. 2015 | | |
| *D. distichum* (C.Presl) Rchb.f. | SI | Pinheiro et al. 2015 | | |
| *D. dixanthum* Rchb.f. | SC | Pinheiro et al. 2015 | N | Jia, Huang 2021 |
| *D. draconis,* Rchb.f. | SI | Niu et al. 2018 | | |
| *D. ellipsophyllum* Tang & F.T.Wang | SI | Niu et al. 2018 | NL | Jia, Huang 2021 |
| | | Pinheiro et al. 2015 | | |
| *D. exile* Schltr. | SC | Niu et al. 2018 | | |
| | | Pinheiro et al. 2015 | | |
| *D. falconeri* Hook. | SI | Niu et al. 2018 | | |
| | | Pinheiro et al. 2015 | | |
| *D. farmeri* Paxton | SI | Niu et al. 2018 | N | Own research |
| | | Pinheiro et al. 2015 | | |
| *D. fimbriatum* Hook. | SC | Niu et al. 2018 | N | Jia, Huang 2021 |
| *D. findlayanum* | SC | Niu et al. 2018 | | |
| *D. formosum* Roxb. Ex Lindl. | SC | Niu et al. 2018 | | |
| *D. friedericksianum* Rchb.f. | SI | Niu et al. 2018 | N | Own research |
| *D. gibsonii* Paxton | SC | Niu et al. 2018 | N | Jia, Huang 2021 |
| *D. glomeratum* H.J.Veitch ex Rob. | - | - | N | Own research |
| *D. gratiosissimum* Rchb.f. | SI | Niu et al. 2018 | N | Jia, Huang 2021 |
| | | Pinheiro et al. 2015 | | |
| *D. griffithianum* Lindl. | SI | Pinheiro et al. 2015 | | |
| *D. hainanense* Rolfe | SC | Niu et al. 2018 | | |
| *D. hancockii* Rolfe | SI | Niu et al. 2018 | N | Jia, Huang 2021 |
| *D. harveyanum* Rchb.f. | SI | Niu et al. 2018 | | |
| *D. hendersonii* A.D.Hawkes & A.H.Heller | SI | Pinheiro et al. 2015 | | |
| *D. hercoglossum* Rchb.f. | SC | Niu et al. 2018 | NL | Jia, Huang 2021 |
| | | Pinheiro et al. 2015 | | |
| *D. heterocarpum* Wall. ex Lindl. | SC | Niu et al. 2018 | | |
| | | Pinheiro et al. 2015 | | |
| *D. indivisum* (Blume) Miq. | SI | Pinheiro et al. 2015 | | |
| *D. infundibulum* Lindl. | SC | Niu et al. 2018 | NL | Jia, Huang 2021 |
| *D. jenkinsii* Wall. Ex Lindl. | SI | Niu et al. 2018 | | |
| *D. keithii* Ridl. | SI | Pinheiro et al. 2015 | | |
| *D. kingianum* Bidwill ex Lindl. | SI | Pinheiro et al. 2015 | N | Own research |

| | | | | |
|---|---|---|---|---|
| *D. leonis* (Lindl.) Rchb.f. | SI | Niu et al. 2018 | | |
| | | Pinheiro et al. 2015 | | |
| *D. leptocladum* Hayata | SI | Pinheiro et al. 2015 | | |
| *D. linawianum* Rchb.f. | SC | Niu et al. 2018 | NL | Jia, Huang 2021 |
| *D. lindleyi* Steud. | SI | Niu et al. 2018 | | |
| | | Pinheiro et al. 2015 | | |
| *D. linguella* Rchb.f. | SI | Pinheiro et al. 2015 | | |
| *D. lituiflorum* Lindl. | SC | Niu et al. 2018 | | |
| *D. loddigesii* Rolfe | SC | Niu et al. 2018 | N | Jia, Huang 2021 |
| | | Pinheiro et al. 2015 | | |
| *D. lohohense* Tang & F.T.Wang | - | - | N | Jia, Huang 2021 |
| *D. longicornu* Lindl. | SI | Niu et al. 2018 | NL | Jia, Huang 2021 |
| *D. maccarthiae* Thwaites | - | - | | |
| *D. macrophyllum* A.Rich. | SC | Pinheiro et al. 2015 | N | Own research |
| *D. macrostachyum* Lindl. | SC | Pinheiro et al. 2015 | | |
| *D. modestum* Rchb.f. | SI | Own research | N | Own research |
| *D. moniliforme* (L.) Sw. | SI | Niu et al. 2018 | NL | Jia, Huang 2021 |
| *D. monophyllum* F.Muell. | SI | Bartareau, 1995 | NL | Bartareau, 1995 |
| *D. moschatum* (Banks) Sw. | SI | Niu et al. 2018 | | |
| | | Pinheiro et al. 2015 | | |
| *D. nathanielis* Rchb.f. | SI | Pinheiro et al. 2015 | | |
| *D. nobile* Lindl. | SC | Pinheiro et al. 2015 | NL | Jia, Huang 2021 |
| *D. officinale* Kimura & Migo | SC | Niu et al. 2018 | NL | Jia, Huang 2021 |
| *D. pachyglossum* C.S.P.Parish & Rchb.f. | SI | Pinheiro et al. 2015 | | |
| *D. pachyphyllum* (Kuntze) Bakh.f. | SI | Niu et al. 2018 | | |
| | | Pinheiro et al. 2015 | | |
| *D. panduriferum* Hook.f. | SI | Pinheiro et al. 2015 | | |
| *D. parcum* Rchb.f. | SI | Pinheiro et al. 2015 | | |
| *D.parishii* H. Low | SI | Pinheiro et al. 2015 | N | Jia, Huang 2021 |
| *D. pendulum* Roxb. | SC | Niu et al. 2018 | | |
| | | Pinheiro et al. 2015 | | |
| *D. polyanthum* Wall. Ex Lindl. | SC | Niu et al. 2018 | | |
| *D. polytrichum* Ames | - | - | N | Own research |
| *D. porphyrochilum* Lindl. | SI | Niu et al. 2018 | | |
| *D. primulinum* Lindl. | SI | Pinheiro et al. 2015 | N | Jia, Huang 2021 |
| *D. pulchellum* Roxb. ex Lindl. | SI | Niu et al. 2018 | | |
| | | Pinheiro et al. 2015 | | |
| *D. salaccense* (Blume) Lindl. | SC | Pinheiro et al. 2015 | | |
| *D. scoriarum* W.W. Sm. | SC | Niu et al. 2018 | NL | Jia, Huang 2021 |
| *D. secundum* (Blume) Lindl. ex Wall. | SI | Niu et al. 2018 | N | Own research |
| | | Pinheiro et al. 2015 | | |
| *D. senile* C.S.P.Parish & Rchb.f. | SI | Pinheiro et al. 2015 | | |

| | | | | |
|---|---|---|---|---|
| *D. setifolium* Ridl. | SI | Pinheiro et al. 2015 | | |
| *D. signatum* Rchb.f. | SI | Niu et al. 2018 | NL | Jia, Huang 2021 |
| *D. spatella* Rchb.f. | SI | Niu et al. 2018 | | |
| *D. speciosum* Sm. | SI | Pinheiro et al. 2015 | NL | Jia, Huang 2021 |
| *D. spectabile* (Blume) Miq. | SC | Pinheiro et al. 2015 | | |
| *D. stratiotes* Rchb.f. | SC | Pinheiro et al. 2015 | | |
| *D. strongylanthum* Rchb.f. | SI | Niu et al. 2018 | | |
| *D. stuposum* Lindl. | SI | Niu et al. 2018 | N | Jia, Huang 2021 |
| *D. subulatum* (Blume) Lindl. | SI | Pinheiro et al. 2015 | | |
| *D. sulcatum* Lindl. | SC | Pinheiro et al. 2015 | N | Jia, Huang 2021 |
| *D. terminale* C.S.P.Parish & Rchb.f. | SC | Niu et al. 2018 | | |
| *D. thyrsiflorum* B.S. Williams | SI | Niu et al. 2018<br>Pinheiro et al. 2015 | N | Jia, Huang 2021 |
| *D. tortile* Lindl. | SC | Pinheiro et al. 2015 | | |
| *D. trantuanii* Perner & X.N.Dang | SC | Niu et al. 2018 | | |
| *D. trigonopus* Rchb.f. | SC | Niu et al. 2018 | | |
| *D. trinervium* Ridl. | - | - | N | Own research |
| *D. unicum* Seidenf. | SC | Niu et al. 2018 | | |
| *D. virgineum* Rchb.f. | SI | Niu et al. 2018<br>Pinheiro et al. 2015 | | |
| *D. wardianum* R.Warner | SC | Niu et al. 2018<br>Pinheiro et al. 2015 | | |

i – SC - self-compatible species; SI - self-incompatible

ii – N – nectariferous; NL– nectarless
